# Ascending aortic aneurysm caused by *Mycobacterium tuberculosis*

**DOI:** 10.1186/s13104-015-1667-x

**Published:** 2015-11-09

**Authors:** Upul Pathirana, Saman Kularatne, Suneth Karunaratne, Gamini Ranasinghe, Janakie Fernando

**Affiliations:** Department of Respiratory Medicine, National Hospital for Respiratory Diseases, Welisara, Sri Lanka; Department of Cardiology, National Hospital of Sri Lanka, Colombo, Sri Lanka; Department of Cardiothoracic Surgery, National Hospital of Sri Lanka, Colombo, Sri Lanka; Department of Pathology, National Hospital of Sri Lanka, Colombo, Sri Lanka

**Keywords:** Aortic aneurysm, Tuberculous aortitis, Aortic valve replacement, Aortic root replacement

## Abstract

**Background:**

Tuberculous aortitis is an unusual presentation of a common disease in Sri Lanka. There were no reported cases of tuberculous aortitis from Sri Lanka. Here we report a case of a 40-year-old woman who developed an ascending aortic aneurysm with severe aortic regurgitation caused by *Mycobacterium tuberculosis*.

**Case presentation:**

A 40-year-old Sri Lankan female who presented with exertional breathlessness (NYHA II) and weight loss for 4 weeks duration was found to have collapsing pulse and early diastolic murmur at left sternal edge. Transthoracic and transesophageal echocardiogram showed ascending aortic aneurysm with severe aortic regurgitation. Computed tomographic aortography confirmed the diagnosis of aneurysmal dilatation of the ascending aorta. She underwent successful aortic valve replacement and aortic root replacement. The final diagnosis of tuberculous aortitis was made on the basis of macroscopic appearance of inflammation and microscopic confirmation of caseating granuloma. She made a good clinical recovery with category 1 antituberculous chemotherapy.

**Conclusions:**

Although most cases of aortitis are non-infectious in Sri Lanka, an infectious etiology must be considered in the differential diagnosis because therapeutic approaches differ widely. Tuberculous aortitis may be under diagnosed in Sri Lanka, a country with intermediate tuberculosis burden, as the histological or microbiological diagnosis is not possible in most cases. The clinical and radiological diagnostic criteria for tuberculous aortitis need to be set out in case of aneurysmal aortic disease in the absence of apparent etiology.

## Background

The diagnosis of rare infectious aortitis should be made accurately as the treatment strategy differ from that of more common non-infectious aortitis such as giant cell arteritis (GCA) and Takayasu arteritis. Bacterial aortitis is most commonly associated with Salmonella, *Staphylococcal* species and *Streptococcus pneumoniae* which seed the aortic wall with preexisting pathology via the vasa vasorum [1]. Mycotic aneurysm caused by *Mycobacterium tuberculosis* is a rare disease entity, with involvement of the aortic root being exceedingly rare [2]. Aortic involvement is commonly due to direct seeding of aorta by adjacent infected tissues such as mediastinal lymphadenitis or pulmonary lesions though rarely it may be blood bourn [3]. The diagnosis is difficult to establish as it mimics Takayasu arteritis in addition to its rarity. However early diagnosis is crucial to prevent rupture and associated mortality if left untreated. Thus, the diagnosis of tuberculous aortitis (TA) should be suspected in any patient presenting with aortitis or aortic aneurysm who has evidence of pulmonary or extra-pulmonary tuberculosis in other organs at present or in the past. However the definitive diagnosis is most often established by histology in surgical specimens.

## Case presentation

A 40-year-old Sri Lankan female presented with exertional breathlessness (NYHA II) and weight loss for 4 weeks duration. There was no cough, hemoptysis, evening pyrexia or night sweats and no history of limb claudication. Her past history was negative for connective tissue disorders, vasculitis or tuberculosis. The family history was unremarkable. General examination did not reveal any abnormal physical signs. She had collapsing pulse and blood pressure of 110/40 mmHg in both arms. Early diastolic murmur was heard in both left and right sternal edge. The lung bases were clear and rest of the systemic examination was normal.

Chest x-ray showed smooth dilatation of the ascending aorta and walls of it are not parallel to each other. Transthoracic and transeseophageal echocardiogram showed ascending aortic aneurysm (maximum diameter of 54 mm) with severe aortic regurgitation. Cardiac chamber dimensions were within normal limits and left ventricular ejection fraction was normal. Computed tomographic (CT) aortography confirmed the dilatation of aortic root and ascending aorta without dissection or leaking. Acute phase reactants were slightly elevated (erythrocyte sedimentation rate 50 mm 1st hour, C-reactive protein 18 mg/dL). Venereal Disease Research Laboratory test (VDRL) was non-reactive. Antinuclear antibody was negative.

**Fig. 1:**
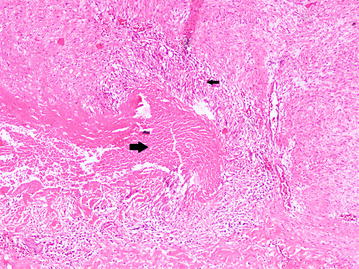
Photomicrograph of of excised specimen of aortic wall. Well-formed granuloma (*small black arrow*) and central caseous necrosis (*large black arrow*). (Hematoxylin and eosin stain, ×100)

**Fig. 2:**
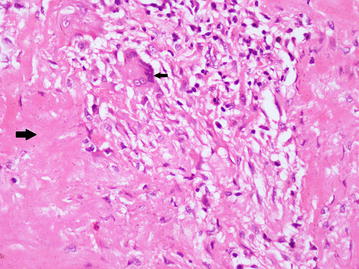
Photomicrograph of excised specimen of aortic wall. Foci of histiocyte collection, some admixed plasma cells and caseous necrosis (*large black arrow*). Langerhans type giant cells are also seen (*small black arrow*). (Hematoxylin and eosin stain, ×400)

Cardiology team proceeded their further evaluation with pre-operative coronary angiogram which showed normal epicardial coronary arteries. Aortic valve and root replacement was recommended by the cardiologist. Cardiothoracic surgical team carried out the aortic valve replacement (AVR) with 19 mm bileaflet St Jude Medical mechanical valve and aortic root replacement with 30 mm Albo graft. Surgeon noted evidence of aortitis with external inflammatory adhesions during the surgery. Post-operative period was uneventful. Warfarin therapy was started while closely monitoring the INR and it was kept between 2 and 3. Histological examination of aortic wall revealed granulomatous aortitis with caseous necrosis (Figs. 1, 2) even though it did not show acid fast bacilli (AFB) on Ziehl–Neelsen staining (ZNS).

The final diagnosis of ascending aortic aneurysm with severe aortic regurgitation due to tuberculous aortitis was made on the basis of clinical, imaging and histological findings. She was treated with category one antituberculous therapy (ATT) consisting of 3 fixed dose combination tablets per day each containing rifampicin 150 mg, isoniazid 75 mg, pyrazinamide 400 mg and ethambutol 275 mg (RHZE). Frequent dose adjustment of warfarin was required during ATT. Continuation phase of ATT was extended for up to 10 months with rifampicin and isoniazid as in the case of disseminated tuberculosis. She made a good clinical recovery with improvement of her symptoms and follow up image showed functioning aortic prosthesis while maintaining INR in therapeutic range.

## Discussion

Tuberculous aortitis is a very rare form of extra-pulmonary tuberculosis. It can be caused by direct extension of Mycobacterium tuberculosis from an adjacent focus of infection or by hematogenous spread. Close proximity of distal aortic arch and the descending aorta to a specific group of mediastinal lymph nodes may explain it being the commonest site for tuberculous aortitis, but exceptionally ascending aorta is affected as in this case [4]. The spectrum of clinical presentation varies widely. More commonly, tuberculous aortitis is associated with aneurysmal dilation which can present with acute aortic syndromes such as rupture or dissection or it may be an incidental finding on chest imaging [5]. Stenotic lesions involving the aorta (acquired coarctation) or renal artery stenosis can present with hypertension [6].

Many infectious and non-infectious etiologies are associated with aortitis. Negative VDRL test makes the syphilitic aortitis less likely as a cause for ascending aortic aneurysm in this patient. She did not have any typical clinical features or radiological criteria of Takayasu arteritis. Giant cell arteritis was not a consideration in this case as it affects individuals older than 50 years of age [7]. Absence of clinical features and negative autoimmune markers excluded the possibility of other rare causes like vasculitis and connective tissue disorders.

Tuberculous arteritis is an important consideration in this case as new case finding rate of all tuberculous diseases (pulmonary and extra-pulmonary) are 9000–10,000 in Sri Lankan annually [8]. The diagnosis of tuberculous aortitis is very challenging and the diagnosis without histology is further challenging. Active tuberculosis in any other organ or past history of tuberculosis may direct the clinician towards this very rare diagnosis. However she did not have any evidence of active or past tuberculosis in any other organ. Computed tomography in case of aortic inflammation may show wall thickening and peri-aortic inflammation as it is described in other form of aortitis, but it is less sensitive and not evident in her CT films [9]. In contrast to CT, magnetic resonance angiography (MRA) is sensitive to detect wall thickening and edema in case of aortitis [10].

The definitive diagnosis was ultimately established by histology of the surgical specimen which confirmed it as granulomatous aortitis with evidence of caseous necrosis. Although positive AFB on ZNS strongly favor the diagnosis of tuberculous aortitis, negative test does not exclude the diagnosis as the sensitivity of this test is very low. Granulomatous aortitis with caseous necrosis more favor the diagnosis of tuberculous aortitis even though no evidence of tuberculosis in another site.

She complied with Category one ATT without any drug induced complications, but warfarin therapy to maintain INR in therapeutic range (2–3) needed frequent monitoring and dose adjustments as some of the anti-tuberculous drugs are known liver enzyme inducers. Since this form of tuberculosis is indicative of disseminated disease [6], we decided to extend continuation phase for up to 10 months.

## Conclusions

Infectious aortitis like tuberculosis should be considered as an etiology for aortic aneurysm. Diagnostic criteria without histological specimens are defined for non-infectious aortitis like Takayasu arteritis and GCA. Surgical biopsy or specimens are often needed for the confirmation of infectious etiology such as tuberculous aortitis though it is not possible in all cases. Noninvasive imaging techniques like MRA are emerging investigation modalities to detect aortic wall inflammation and peri-aortitis. High degree of suspicion in an appropriate clinical setting in combination with imaging may justify the treatment with ATT. Development of diagnostic criteria for tuberculous aortitis based on the clinical findings and imaging may be a future research area.

## Consent

Written informed consent was obtained from the patient for publication of this case report and any accompanying images.

## Abbreviations

GCA: giant cell arteritis
CT: computed tomography
AVR: aortic valve replacement
AFB: acid fast bacilli
ZNS: Ziehl–Neelsen staining
ATT: anti-tuberculous therapy
MRA: magnetic resonance angiography
